# Purulent Endometritis during Pregnancy

**Published:** 1885-08

**Authors:** 


### Purulent Endometritis During Pregnancy.

J. Donat in the *Archiv für Gynäk* reports a case of purulent endometritis occurring in pregnancy, which is unique.

Healthy child; born at term; mother healthy; no history of venereal disease.

Between the amnion and chorion, in the decidua vera, and in the decidua serotina, there was purulent infiltration.

No gonococci were found. It was evident that the purulent process began in the decidua vera and serotina.

The reporter has failed to find a case of purulent endometritis in pregnancy anywhere in gynæcological literature.

